# Aesthetic perception of single implants placed in the anterior zone. A cross-sectional study

**DOI:** 10.4317/medoral.21155

**Published:** 2016-03-31

**Authors:** Genís Burgueño-Barris, Berta Cortés-Acha, Rui Figueiredo, Eduard Valmaseda-Castellón

**Affiliations:** 1 DDS. Specialty registrar. Master in Oral Surgery and Implantology. Faculty of Dentistry of the University of Barcelona, Spain; 2 DDS. PhD. Associate lecturer. Oral Surgery and Implantology Department, Faculty of Dentistry of the University of Barcelona. Researcher at the IDIBELL Institute, Barcelona, Spain; 3 DDS. PhD. Tenured lecturer. Oral Surgery and Implantology Department, Faculty of Dentistry of the University of Barcelona. Researcher at the IDIBELL Institute, Barcelona, Spain

## Abstract

**Background:**

Several aesthetic indexes have been described to assess implant aesthetics. The aim of this study was to compare the aesthetic assessment made by dental professionals and students of single-tooth implants placed in the upper incisors.

**Material and Methods:**

A cross-sectional survey study using a subjective questionnaire to assess the aesthetics in 3 implant supported single-tooth cases in the anterior maxilla was performed. The interviewed subjects were divided into 4 groups: dentists with experience in implant treatment, dentists without experience in implants and 3rd and 5th year dental students. The questionnaire consisted of 2 visual analogue scales (VAS) to evaluate aesthetics, the pink esthetic score (PES), the white esthetic score (WES) and the simplified papilla index (PI).

**Results:**

One-hundred dentists and one-hundred dental students filled the aesthetic assessment questionnaire. The results showed that the subjects were more critical than reference values, specially concerning prosthetic issues. The differences between groups were more obvious in the case with the best result. On the other hand, few differences were detected in the remaining cases. Regarding soft tissue and crown features, experienced dentists in implant dentistry were the most demanding. Cronbach’s Alpha showed values ≥ 0,8 in the questionnaire in every case, which indicates an adequate reliability.

**Conclusions:**

Dentists and dental students have different opinions when assessing aesthetics of single tooth implant supported cases. Experience and area of expertise seem to influence the evaluation of aesthetics in the anterior region.

**Key words:**Dental implant, anterior area, aesthetics.

## Introduction

The rehabilitation of single tooth gaps in the anterior zone with dental implants has become a common treatment due to its high predictability. It has been reported that implant survival and success rates in the aesthetic zone are similar to those placed in other segments (1). Nevertheless, aesthetic demands have increased and rehabilitation of anterior teeth is still a challenge for surgeons and prosthodontists (1-3). Even so, patients have a high overall satisfaction with implant treatment in the anterior maxilla (4-6).

The most commonly used index to assess aesthetics has been proposed by Belser *et al.* (2009) (1). This index has been validated in several studies that assessed implant restorations (1,7-13). On the other hand, the papilla index (PI) described by Jemt (14) or the pink esthetic score (PES), stress the importance of the papilla adjacent to the implant. Indeed, the presence and form of the interdental papilla is a key feature in the soft tissue architecture. However, obtaining a well designed papilla around implants remains a challenge for the dental professional despite the great deal of techniques that have been described for its preservation, manipulation and reconstruction (15). Another matter of concern are the differences between dentists and patients perceptions’ of both soft and hard tissues, especially when assessing single implant restorations placed in the aesthetic area.

Patient’s perception has usually been evaluated with questionnaires or visual analogue scales (VAS) (1). It has been reported that the preoperative situation and expectations play a significant role in the final judgment of the restoration (11,16). When the preoperative situation is compromised and patient’s expectations are realistic, a poor outcome from a clinician’s point of view can be perceived by the patient as a successful treatment (11,16). However, it is still unclear whether clinicians and patients have the same aesthetic demands. Indeed, less experienced professionals (i.e. dental students) could be less critical than dentists because they might have different perspectives on implant aesthetics. Therefore, future research should be conducted to establish well-defined parameters to measure aesthetics in implant dentistry (17-20).

The aim of this study was to compare the aesthetic assessment of single-tooth implants in the upper incisors, by different dental professionals (dentists with and without experience in implant dentistry) and dental students.

## Material and Methods

A cross-sectional survey using a subjective aesthetic questionnaire was performed. STARD guidelines were used to improve the accuracy, avoid biases and evaluate its validity (21).

- Sample

Professionals were classified according to their professional background into 4 groups.

• Dentists with experience in implant dentistry: Clinicians with exclusive or preferential dedication to implant therapy, with postgraduate studies in implantology and a minimum of 5 years of clinical experience in the above-mentioned field. This group was subdivided into: Oral surgeons, periodontists and prosthodontists.

• Dentists without experience in implantology: Clinicians with at least 5 years of experience in dental procedures, but with no dedication to implant therapy. This group was also divided into: general dentists, endodontists, paediatric dentists and orthodontists.

• Third and 5th-year students of the School of Dentistry of University of Barcelona.

- Data collection

The dentists were recruited in the Dental Hospital of the University of Barcelona (usually members of clinical teaching staff) and also in private dental practices from the Barcelona area. Fifty dentists per group were selected after a power analysis for the difference between 2 proportions, with alpha error=0.05, beta error=0.2, difference of proportions = 0.2 using G*Power (22).

Data collection entailed a personal interview to solve any doubt when answering the questionnaires.

- Aesthetic assessment 

**Figure 1:**
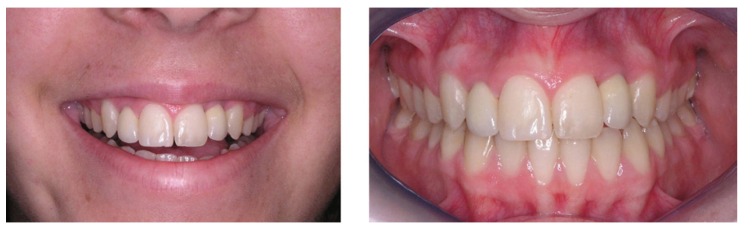
Case 1 (Implant in left maxillary lateral incisor).

**Figure 2:**
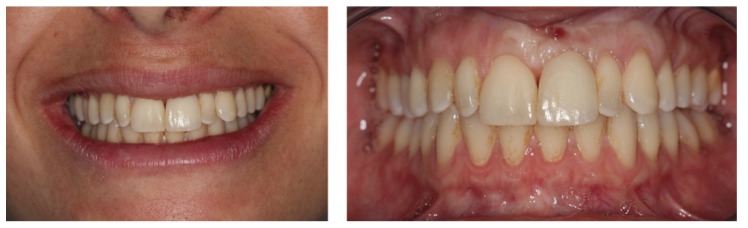
Case 2 (Implant in left maxillary central incisor).

**Figure 3:**
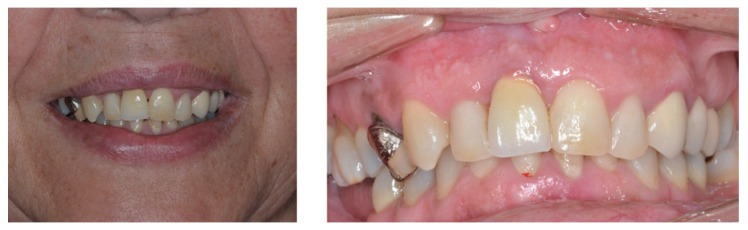
Case 3 (Implant in right maxillary central incisor).

Every participant anonymously filled 3 questionnaires (one for each case) regarding the evaluation of patients with single implant restorations in the anterior maxilla. Two frontal photos (an intraoral view with lip retraction and a picture of the patient’s smile) were provided (Figs. 1-3). Cases were selected so that the first case had the best overall aesthetic result and last patient had the worst outcome, without being an obvious aesthetic failure. The patients’ identity remained confidential and an informed consent was signed allowing using the photos. Approval by an Institutional Review Board was not required since this cross-sectional study was based in a sample of dental professionals and not patients.

The questionnaire included two 10-cm visual analogue scales (VAS) labelled from “better possible result” (10) to “worst possible result” (0) to assess the smile appearance and teeth harmony. Moreover, the survey included the question “Do you know which is the implant supported tooth?”. If the participant replied incorrectly, the researcher showed the correct answer to the dentist or student.

Additionally, the questionnaire included a series of items based on the Pink Esthetic Score / White Esthetic Score (PES/WES) and the reduced version of the papilla index (PI).

Once all the questionnaires were filled, 2 calibrated and experienced dentists established the referenced values after rating the cases using the same form. PES/WES index scores ranged from 0 to 2; and PI results ranged from 0 to 4.

- Statistical analysis

Frequencies, mean values and standard deviations (SD) were calculated. Associations between categorical variables were tested with the Pearson’s Chi-square test. Intraclass correlation coefficient (ICC) was used to analyse the scale variables (VAS questions) and Cronbach’s alpha was used to assess test reliability and internal consistency. All analyses were performed using a computer software (IBM SPSS 22.0®; IBM, Armonk, USA).

Results

A total of 200 interviews were analyzed: fifty 3rd-year dental students, fifty 5th-year dental students and 100 dentists (50 with experience and 50 without experience in implant dentistry). Expertise areas of the practitioners were as follows: 7 endodontists, 13 general dentist, 13 orthodontists, 9 paediatric dentists, 8 periodontist, 24 prosthodontists and 26 oral surgeons.

Cronbach’s Alpha showed values ≥ 0,8 in all case (all 14 questions and without stratification for the 4 groups) ( Table 1). Therefore, items of the questionnaire items showed a good correlation, thus indicating a good reliability.

**Table 1:**
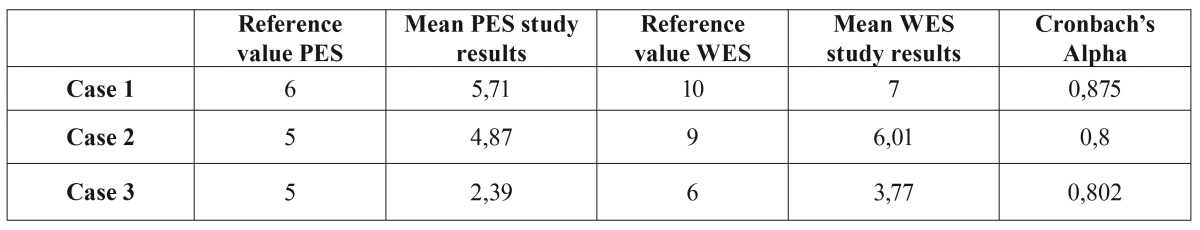
Reference value PES/WES compared to mean study results and Cronbach’s Alpha of each case.

 Table 1 compares the reference values scored by two calibrated experts to the mean value of the answered questionnaires. This data show that there was only agreement with the reference values in the PES of cases 1 and 2. The reference value of the WES questions given by the calibrated experts was considerably higher than the mean results of the participants in all cases, which indicates that the respondents were quite critical, especially in the prosthetic aspects.

The correlation between the results of the 2 VAS (smile appearance and teeth harmony) and the intraclass correlation coefficient (ICC) was higher in all cases in the dentists with experience in the implant dentistry field. In case 1, which received the highest score from the 2 calibrated dentists, correlation values were poorer (<0,8), especially in the professionals without experience (0,465).

When asked to identify the crown, dental students (especially 3rd year students) failed more often. Indeed, significant differences were found in this variable for the first 2 cases, but not for the case with the worst aesthetic result.

On the other hand, there were significant differences among respondents in the 4 different groups regarding papilla, soft tissue and crown features in the first case ( Table 2). Few differences were detected in PES (without papilla questions) in case 3 and in WES questions in case 2. Therefore, the differences between groups were more evident in case 1, which the researchers considered as having the best result.

**Table 2:**
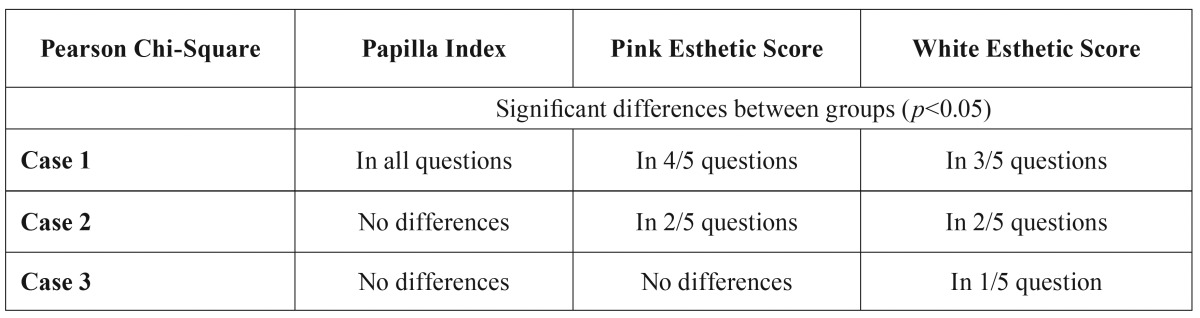
Differences between groups regarding Papilla Index, Pink Esthetic Score and White Esthetic Score.

 Table 3, Table 4 show the percentage of the worst results on papilla questions (2 first questions of PES and questions of PI) in each group.  Table 3 shows that 5th-year dental students were very critical evaluating the papillas, especially in cases 2 and 3. In the group of dentists, the endodontists were the most demanding regarding the papilla anatomy ( Table 4).

**Table 3:**
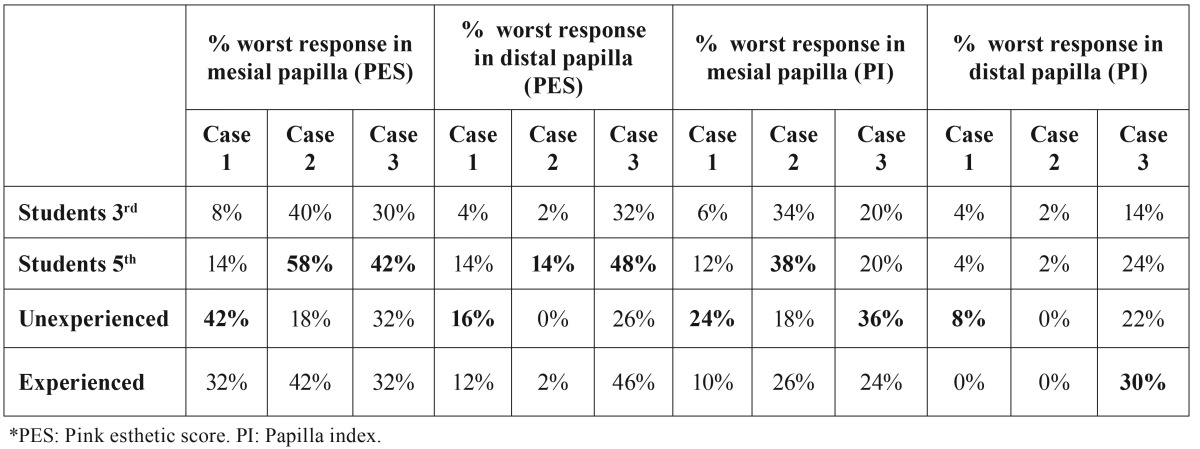
Percentage of worst response in papilla variables. In bold: worst result of each question. Regarding papilla evaluation, 5th year dental students and dentists without experience were more critical when compared to clinicians with experience in implant dentistry.

**Table 4:**
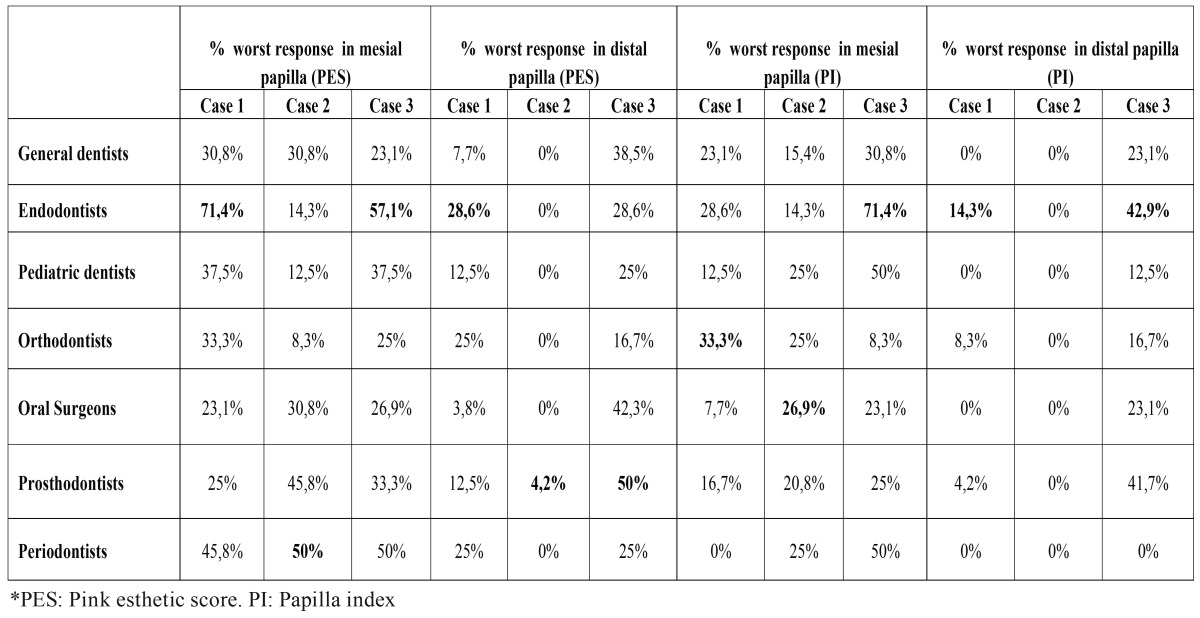
Percentage of worst response in papilla variables divided in areas of knowledge. In bold: worst result in each question. In papilla questions, endodoncists were the most harsh.

## Discussion

Several objective classifications have been created to assess implant aesthetics. Some of them, like the Pink Esthetic Score/White Esthetic Score and Papilla Index have shown a good reproducibility and validity (1,7-14). Nevertheless, the study results yielded a lack of correlation between dentists. These discrepancies depended on professional’s experience and expertise area, which clearly jeopardizes the generalization of the above-mentioned classifications.

In our sample, clinicians criticized soft tissue parameters but were generally stricter when assessing the crown. Therefore, dentists seemed to be very critical with prosthodontic parameters. This difference may be related to the fact that professionals are more used to assess the aesthetics of crowns in clinical practice because they are able to change shape, colour or texture by sending the crown back to the dental technician, while soft tissues are more difficult to modify.

As expected, dentists with experience in implantology made a stricter appraisal of the best aesthetic outcome case. On the remaining 2 cases, the results were similar across the groups, but there was a lack of agreement between professionals.

There are very few studies that compare the aesthetic assessment of dental implant cases, made by different groups of dentists. Some reports (12,23-25). showed that orthodontists were more critical than general dentists, and that prosthodontists were generally very strict with the WES scores (12). This might be explained by the daily activities of these professionals, since patients usually demand a high aesthetic result after a prosthetic or orthodontical treatment. Indeed, these groups of dentists tend to focus their attention to details such as crown features or symmetry and proportions. On the other hand, general dentists might be more indifferent to this kind of details. Indeed, our results indicate the need to improve the communication between specialties, so new classifications with a better external validity can be implemented.

The present study points out that important differences can be found between diverse profiles of dentists, especially in very aesthetic cases. Thus, it is highly likely that even worst discrepancies can be found between dentists and patients. This fact is extremely important since patients’ aesthetic assessment might be strictly related with their expectations, and therefore, with the treatment success. Most studies on this issue conclude that dentists are more critical than patients regarding both soft tissue and crown anatomy evaluation (1,3-5-7,8,11,13,14,17,18,26-29). Papillas are usually considered a key factor to dentists, and many techniques have been described to improve the shape and size of interproximal soft tissues. In fact, many times, clinicians indicate additional surgical procedures or the placement of provisional crowns just to improve the shape of this structure. However, an important proportion of patients do not seem to pay much attention to this detail. Therefore, in our opinion, it is mandatory to discuss the need of these procedures with the patients, since they increase morbidity, treatment time and costs, but might not improve the final treatment outcome, from a patient’s perspective (3,26). Further research on this topic should be made.

To summarize, dental professionals and students have different criteria when assessing the aesthetics of single tooth implant supported cases. Experience and area of expertise seem to influence the aesthetics evaluation of the anterior region, especially in cases with good results. The current classifications do not seem to allow generalization of the aesthetic results of single implant supported crowns in the maxilla.
